# Supplementary material for: MetaQTL: a package of new computational methods for the meta-analysis of QTL mapping experiments
Source: BMC Bioinformatics. 2007 Feb 8;8:49. doi: 10.1186/1471-2105-8-49 (PMC1808479; doi:10.1186/1471-2105-8-49)
Supplement: Additional File 7 — MetaQTL Package : jar file and tutorial. This Zip archive contains both the MetaQTL JAR file and the files of the tutorial. [file 1471-2105-8-49-S7.zip › org.inra.metaqtl/doc/org/metaqtl/graph/ChromGraph.html]

ChromGraph


|  |  |  |
| --- | --- | --- |
| **Overview** \| **Package** \| **Class** \| **Use** \| **Tree** \| **Deprecated** \| **Index** \| **Help** | |  |
| **PREV CLASS**   **NEXT CLASS** | **FRAMES**    **NO FRAMES**     **All Classes** |  |
| SUMMARY: NESTED \| FIELD \| CONSTR \| METHOD | DETAIL: FIELD \| CONSTR \| METHOD |  |


---


## org.metaqtl.graph Class ChromGraph

```
java.lang.Object
  org.metaqtl.graph.ChromGraph
```

---

``` public final class ChromGraph extends java.lang.Object ```

A `ChromGraph` allows to link together
a `Chromosome` and a `QtlPartition`
in order to be depicted into a graphic.

---

| **Field Summary** | |
| --- | --- |
| `Chromosome` | `chromosome`             The chromosome to draw |
| `QtlPartition` | `qtlPart`             The Qtl partition |


**Constructor Summary**

`ChromGraph()`


**Method Summary**

**Methods inherited from class java.lang.Object**

`clone, equals, finalize, getClass, hashCode, notify, notifyAll, toString, wait, wait, wait`

**Field Detail**

### chromosome

```
public Chromosome chromosome
```

:   The chromosome to draw

---


### qtlPart

```
public QtlPartition qtlPart
```

:   The Qtl partition


**Constructor Detail**

### ChromGraph

```
public ChromGraph()
```


---


|  |  |  |
| --- | --- | --- |
| **Overview** \| **Package** \| **Class** \| **Use** \| **Tree** \| **Deprecated** \| **Index** \| **Help** | |  |
| **PREV CLASS**   **NEXT CLASS** | **FRAMES**    **NO FRAMES**     **All Classes** |  |
| SUMMARY: NESTED \| FIELD \| CONSTR \| METHOD | DETAIL: FIELD \| CONSTR \| METHOD |  |


---
